# Element cycling by environmental viruses

**DOI:** 10.1093/nsr/nwae459

**Published:** 2024-12-23

**Authors:** Di Tong, Jianming Xu

**Affiliations:** Institute of Soil and Water Resources and Environmental Science, College of Environmental and Resource Sciences, Zhejiang University, China; Zhejiang Provincial Key Laboratory of Agricultural Resources and Environment, Zhejiang University, China; Institute of Soil and Water Resources and Environmental Science, College of Environmental and Resource Sciences, Zhejiang University, China; Zhejiang Provincial Key Laboratory of Agricultural Resources and Environment, Zhejiang University, China

Viruses, the most prevalent biological entities on Earth with an order of magnitude greater abundance than prokaryotes, can infect all domains of life with extraordinary genomes comprising DNA and RNA [1,2]. The diverse lifestyles of viruses (i.e. killing hosts through viral lysing (lytic cycle) versus helping hosts survive in harsh environments via auxiliary metabolic genes (AMGs) (lysogenic cycle)) make virus interactions with organisms and their subsequent impacts on biogeochemical cycling intricate and diverse. Despite the growing number of studies on viruses, the majority of research is focused on mining viral genomic resources, deciphering global virus distributions, or speculating on viral effects on biogeochemistry through metagenomic, meta-transcriptomic, and viromic approaches [3,4]. The impact of viruses on biogeochemical cycling remains poorly understood, particularly with regard to AMGs. There is currently no evidence that AMGs function in real-world environments, let alone quantifying their effects on ecological functions. This highlights a new research frontier exploring the quantification of virus impacts on elemental cycling and developing an understanding of how viruses drive organism and elemental interactions with abiotic properties, such as adsorption, desorption, dissolution, precipitation, and redox reactions with minerals. A better understanding of these interactions with biogeochemical cycles will create a new dimension in global environmental cycling of carbon and nutrients.

Nearly 22.5% ± 5.5% of prokaryote cells are lysed by viruses in oceans each day, accounting for the annual

release of 145 Gt of carbon (C), 27.6 Gt of nitrogen (N), and 4.6 Gt of phosphorus (P), thereby having a profound effect on ocean elemental cycling [5]. Especially in bathypelagic ocean environments, virus-induced prokaryote mortality is overwhelmingly far greater than the protistan grazing rate. The released products can be recycled by prokaryotes via a transformation from particulate organic matter (POM) to dissolved organic matter (DOM), which is termed the ‘viral shunt’ mechanism. During the reutilization of lysate DOM by prokaryotes, virus-induced DOM components are converted from a microbially-labile DOM pool to a rather recalcitrant DOM pool [6]. In addition, cell debris and other lytic compounds relatively resistant to microbial degradation may aggregate and be retained in natural environments providing an organic carbon retention mechanism, namely the ‘viral shuttle’ mechanism. The viral shuttle process is much less understood than the viral shunt, which has only been posited in a few review articles. It is thought to act as a carbon sink in oceans; however, the magnitude and elemental composition remain unclear [7].

A greater focus on the viral shuttle mechanism is required to elucidate new directions, such as identifying biomarkers for viruses. For example, viral glycosphingolipids, which act as biomarkers of viral infection in oceans, are a part of a signal transduction pathway regulating the infection of *Emiliania huxleyi* that can induce uninfected cell death [8]. A set of chlorine-iodine–containing metabolites that were induced by viral infection and released during bloom demise is also proposed as the metabolic footprint

for the viral shunt [9]. These studies inspired us to explore other biomarkers that may regulate biogeochemical cycles, and even to measure the amounts of viral residues (similar to the microbial necromass), which will be a landmark achievement in elucidating the role of viruses in driving biogeochemical cycles.

Although both viral shunt and viral shuttle mechanisms have been confirmed to exist in soil environments [10,11], studies directly examining viral effects on soil elemental cycling lag far behind those in marine systems. Importantly, the extent of carbon exchange resulting from the viral shunt mechanism in soils remains unknown, even though the carbon exchange is expected to be much greater in soils than in marine environments owing to the higher viral abundance and frequency of virus-microbe interactions in soils [12]. Moreover, the viral shuttle purported for marine environments does not conceptually represent the process of viral carbon sequestration in soils/sediments, as it emphasizes that viruses aggregate with lysis products and sink from surface waters to the seafloor.

In the soil/sediment environment, there is a limited range of viral movement and a strongly heterogeneous distribution of soil physicochemical properties (e.g. sizes of soil aggregates and pores, irregular distribution of microbial hotspots and activities). Hence, we propose the term ‘viral aggregation’ rather than viral shuttle to more accurately describe the accumulation of lysis products in the soil environment. Therein, lytic cellular products (e.g. lipids, carbohydrates and proteins) are preferentially preserved over oxidized compounds/minerals and more easily encapsulated by soil aggregates. Viruses can also promote the process of microbial anabolism by increasing the carbon-use efficiency of the surviving microbes, altering extracellular enzyme activity and stoichiometry, and accumulating microbial necromass. These interactions further promote the carbon encapsulating effect of the soil microbial carbon pump (MCP).

Lysate DOM can also undergo complex interactions with minerals, including adsorption, occlusion, aggregation, redox reactions and polymerization, suggesting that viruses participate in a myriad of soil mineral carbon pump (MinCP) processes. Virus-driven carbon can flow within complex food webs (e.g. from single to multiple species, or from a single trophic level to the multi-trophic level) owing to the intrinsic viral ability to infect nearly all living organisms [1]. Auxiliary metabolic genes (AMGs) carried by soil viruses can further assist their hosts in metabolizing C, which provides a broader perspective for viruses in C cycling. Thus, the incorporation/integration of soil viruses into existing MCP and MinCP models, namely virus-MCP and virus-MinCP, could substantially improve our understanding of microbial-mediated C cycling as part of the global carbon cycle.

Viruses also influence N-transformation processes related to microbes and significantly affect N-cycling processes (e.g. nitrification and denitrification), through viral lysing or AMGs. Specifically, viruses not only increase the soil NH_4_^+^ concentration by 2–10 fold, they also have the potential to influence host physiology and activity, such as by lysogeny or AMGs for AOA-specific multicopper oxidase occurring during the infection of ammonia-oxidizing archaea (AOA) [13,14].

The C, N and P cycles are intricately coupled during microbial decomposition, and their stoichiometric ratio determines soil microbial catabolism and anabolism. For example, the C/N ratio of soluble cell lysates is very close to that of the cells, and has a high affinity for microbial utilization, which further increases microbial carbon use efficiency [10,15]. In contrast, viral lysis was previously thought to lead to a divergence of C, N and P cycling and a decreased P content given that the C, N, P stoichiometry in bacteria (C:N:P = 60:16:1) is greater than that of viruses (C:N:P = 18:7.3:1), which may further contribute to P limitation for microbes and plants. Much of the P is preferentially incorporated into the DNA/RNA of viral progeny, as predicted by a biophysical scaling model [15]. In contrast to results from bioinformatic analysis, Tong and coworkers posited that viral lysis alleviates microbial P limitation in real-world soils based on the stoichiometric ratio of extracellular enzyme activity [10]. This debate emphasizes that virus-mediated soil elemental cycling remains essentially a black box, highlighting the need for future research across multiple real-world soils.

Greenhouse gas emissions are also influenced by viruses, which is more elusive and complicated. The impact of viruses on microbial respiration is a tradeoff between the rate of bacterial mortality caused by viruses and the utilization of nutrients acquired by survivors, as well as the temperature-dependent effects of viruses on microbial respiration. Intriguingly, viruses can significantly decrease the temperature sensitivity of microbial respiration (Q_10_ values) and maintain Q_10_ values within the bounds of 1–3 [10]. However, the internal mechanisms driving these interactions require further elucidation. As for the metabolism of CH_4_, the related AMGs carried by virus-infecting methanogens and methanotrophs have been expanded from 3 to 24 by mining metagenomes [16]. However, the effect of viruses on net CH_4_ emission or net CH_4_ oxidation has never been documented, nor have the impacts on N_2_O dynamics.

Greenhouse gas emissions notably affect global climate change, and climate change in turn affects how viruses and microbes interact. For example, a higher temperature will stimulate the virus preferred lytic cycle and lead to more virus-host interactions, which further influence virus-driven elemental cycling. Similarly, water content can strongly influence virus lifestyles. Drier soils have more viruses within the lysogenic cycle, which are more resistant to host immunity, whereas wetter soils favor the lytic cycle [17]. Notably, there are no studies examining how viruses affect elemental cycling under climate change.

**Figure 1.**
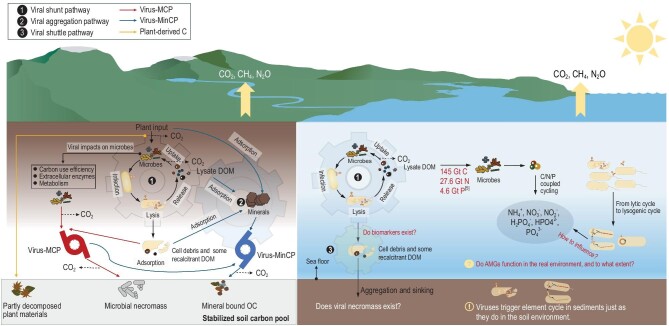
Viral impact on elemental biogeochemical cycles. Viruses in different life cycles can promote elemental cycling (e.g. carbon, nitrogen, and phosphorus) by viral lysing or auxiliary metabolic genes (AMGs) metabolisms. Viruses can increase microbial anabolism (e.g. microbial carbon-use efficiency), which further promotes the carbon encapsulating effect of the microbial carbon pump (MCP). Lysate dissolved organic matter (DOM) can be adsorbed and/or aggregated by minerals, thereby participating in all mineral carbon pumping (MinCP) processes involving carbon sequestration. Moreover, the coupled cycling of carbon, nitrogen, and phosphorus can be strongly influenced by viruses. Determining the impact and extent of these processes requires additional observations and experimental evidence.

Viral impacts on elemental cycles involve many aspects, from carbon input/output fluxes to carbon sequestration mechanisms, and from MCP to MinCP (Fig. 1). Current research is in its infancy and several concepts need to be redefined based on actual environmental conditions, such as the proposed viral aggregation effect in the soil/sediment environment. The emergence of new techniques (e.g. virome characterization, isotope tracers, NEXAFS, FT-ICR-MS and nano-SIMS) could help address essential molecular-level interactions between soil viruses and elemental cycles. For example, new technological advances may allow the identification of viral-mediated polymerized organic C fingerprints in natural environments, analogous to the microbial necromass C fraction. How to quantify the contribution of viruses to elemental cycling is a critical issue that needs to be addressed. For instance, determining an effective method to distinguish soil viruses in the lytic versus lysogenic cycle would fill an important knowledge gap within elemental cycling research. Big data analytics holds great potential for mining soil virus genomic resources and deciphering global virus distributions [18,19]. This could enable a better understanding of viral-mediated elemental fluxes and potential options for regulating elemental footprints (i.e. sequestration versus leaching) on a global scale. In addition, other emerging hot topics (e.g. extreme climatic events (including drought, flooding, freezing and heat), circadian rhythms, and human perturbations) may also offer more intriguing results of virus-driven elemental cycling [20–22]. Overall, experimental and observational studies, from laboratory mesocosms to field experiments, are required to fully disentangle the extent of virus-MCP and virus-MinCP in carbon/elemental cycling and sequestration processes, and the coupled cycles of C, N and P.
